# Cerebellar-Prefrontal Connectivity Predicts Negative Symptom Severity Across the Psychosis Spectrum

**DOI:** 10.1016/j.bpsc.2025.07.013

**Published:** 2025-08-13

**Authors:** Sean A. Yarrell, Sophia H. Blyth, Alexandra B. Moussa-Tooks, Baxter P. Rogers, Anna Huang, Neil D. Woodward, Stephan Heckers, Roscoe O. Brady, Heather Burrell Ward

**Affiliations:** Department of Psychiatry and Behavioral Sciences, Vanderbilt University Medical Center, Nashville, Tennessee (SAY, SHB, BPR, AH, NDW, SH, HBW); Department of Psychological and Brain Sciences, Indiana University, Bloomington, Indiana (ABM-T); Department of Psychiatry, Indiana University School of Medicine, Indianapolis, Indiana (ABM-T); and Department of Psychiatry, Beth Israel Deaconess Medical Center, McLean Hospital, and Harvard Medical School, Boston, Massachusetts (ROB).

## Abstract

**BACKGROUND:**

Negative symptom severity predicts functional outcomes and quality of life in people with psychosis. However, negative symptoms respond poorly to medication, and existing literature has not converged on their neurobiological basis. Previous work in small schizophrenia samples has observed that lower cerebellar–dorsolateral prefrontal cortex (DLPFC) connectivity is associated with higher negative symptom severity and that increasing cerebellar-DLPFC connectivity with neuromodulation reduces negative symptoms. We extended this finding by testing associations between cerebellar-DLPFC connectivity, negative symptoms, and cognitive performance in a large sample of individuals with psychosis.

**METHODS:**

Individuals with psychosis spectrum disorders (*N* = 260) underwent resting-state functional magnetic resonance imaging and clinical characterization using the Positive and Negative Syndrome Scale and the Screen for Cognitive Impairment in Psychiatry. Using a previously identified cerebellar region as a seed, we measured connectivity to the DLPFC and regressed connectivity against negative symptom severity, covarying for age, sex, and scanner. Then, we tested whether cognitive performance indirectly affected the relationship between connectivity and negative symptom severity.

**RESULTS:**

Across the psychosis spectrum, higher cerebellar-DLPFC connectivity was associated with lower negative symptom severity (*r* = −0.17, *p* = .007). This connectivity–negative symptom relationship was not affected by psychosis subtype or duration of illness. Better delayed verbal learning was associated with higher cerebellar-DLPFC connectivity (*r* = 0.13, *p* = .034) and had a significant indirect effect on the relationship between connectivity and negative symptoms.

**CONCLUSIONS:**

Our results extend relationships between cerebellar-DLPFC connectivity, negative symptom severity, and cognitive performance across the psychosis spectrum. Larger neuromodulation studies should test whether increasing cerebellar-DLPFC connectivity reduces negative symptoms in psychotic disorders.

Schizophrenia affects approximately 24 million people worldwide and is characterized by positive and negative symptoms and cognitive impairment (1). Negative symptoms are defined by emotional, social, and psychomotor impairment (2). This includes blunted affect, alogia, anhedonia, asociality, and avolition (3). Negative symptom severity strongly predicts functional outcomes and overall quality of life (4-6). Critically, negative symptoms are often minimally responsive to antipsychotic medication (7,8).

To develop more effective treatments for negative symptoms, previous studies have used neuroimaging to investigate their neurobiological basis. However, existing correlational neuroimaging studies have not yielded consistent findings (9,10). Motor networks including the cerebellum (11) and motor cortex and basal ganglia (12) have been associated with negative symptoms in addition to other regions, such as the striatum (13,14), orbitofrontal cortex (13), supramarginal gyrus (11,15), and superior temporal gyrus (16).

The cerebellum is a complex neural structure that is reciprocally connected with the cerebrum and was more recently shown to contribute to fundamental motor functions, as well as higher-order processes. Recent work has shown that cerebellothalamic pathways operate as a potential anatomical substrate that coordinates neuronal communication between cerebral cortical areas by coordinating the coherence of oscillations (17). Previous studies suggest that hypoconnectivity in these cerebrocerebellar pathways in early psychosis may precede the onset of more serious pathology and psychotic symptomology (18). Interestingly, this coincides with the onset of negative symptoms, which also arise in early prodromal states (8). One critical process is predictive coding, the ability to consolidate incoming sensory information and use it to actively predict upcoming sensory information in one’s environment and to inform decisions and changes in behavior. Predictive coding has often been linked to disturbances in motor control, which can be a notable feature of negative symptom pathology (i.e., withdrawal, motor retardation), but aberrations in predictive processing have also been shown to contribute significantly to higher-level functions (18) such as speech, decision making, and reward processing (i.e., anhedonia) (19), also notable in negative symptom pathology.

A critical study for understanding the role of cerebellar connectivity in negative symptoms was conducted by Brady *et al.* (20), who used a novel, data-driven connectome-wide multivariate analysis that identified a link between lower cerebellar–dorsolateral prefrontal cortex (DLPFC) connectivity and higher negative symptom severity in 44 individuals with schizophrenia or schizoaffective disorder. Next, they applied intermittent theta burst stimulation (iTBS), a form of repetitive transcranial magnetic stimulation (rTMS) associated with excitatory effects, to the cerebellum in 11 individuals with schizophrenia. Compared with sham, in the active stimulation group, increased cerebellar-DLPFC connectivity was associated with greater reductions in negative symptom severity. Following this result, there has been increasing evidence for a cerebellar-DLPFC circuit related to negative symptoms in schizophrenia. Cerebellar–ventral tegmental area (VTA) connectivity has been associated with apathy, a core negative symptom in schizophrenia (19). Moreover, an accelerated iTBS intervention applied to a personalized DLPFC target based on connectivity to the VTA improved negative symptom severity in schizophrenia over sham (21).

Together, evidence from the animal literature on cerebellar control of PFC neurons and TMS studies converge on a cerebellar-DLPFC circuit associated with negative symptom severity in schizophrenia. However, existing research supporting the relationship between cerebellar-DLPFC connectivity and negative symptoms has relied on small samples of individuals with schizophrenia or schizoaffective disorder (*N* = 44 and *N* = 11, respectively) and has not linked these brain-level changes to a cognitive process that can be theoretically linked to negative symptom pathology (20).

Therefore, we sought to test 2 primary hypotheses. First, we determined whether cerebellar-DLPFC connectivity was associated with negative symptom severity across the psychosis spectrum using a large (*N* = 260) sample of individuals with psychosis spectrum disorders collected at an independent site. To maximize reproducibility, we tested connectivity from the exact cerebellar region previously identified by Brady *et al.* (20). We hypothesized that higher cerebellar-DLPFC connectivity would be associated with lower negative symptom severity across psychosis spectrum disorders. Because negative symptoms are predominantly associated with nonaffective psychoses (e.g., schizophrenia), we also investigated whether this connectivity relationship would also be related to negative symptom severity for affective psychoses (e.g., bipolar disorder with psychotic features).

Second, given the role of the cerebellum in cognitive performance in both healthy and psychosis populations (22-26) and a role for predictive coding in negative symptom pathology, we tested whether connectivity was positively related to cognitive performance. Finally, as an exploratory aim to support future interventional work, we conducted an indirect effects analysis to clarify the relationships between cerebellar-DLPFC connectivity, cognitive performance, and negative symptom severity.

## METHODS AND MATERIALS

### Participants

Data came from a repository of 361 individuals with psychotic disorders, with complete neuroimaging and behavioral data, who participated in one of the 3 neuroimaging projects conducted at the Vanderbilt University Medical Center. All studies were approved by the Vanderbilt Institutional Review Board, and all individuals provided written informed consent prior to participation. The Structured Clinical Interview for DSM-IV or DSM-5 was administered to all study participants to confirm diagnoses (27,28) (see the Supplement for details). All participants had 1) a psychosis diagnosis (e.g., schizophrenia) and 2) a psychosis subtype (e.g., nonaffective psychosis). After quality control (see MRI Data Processing), the final sample consisted of 260 individuals with a psychosis spectrum disorder (nonaffective psychosis *n* = 186; affective psychosis *n* = 74) (Tables S1 and S2).

### Assessments

Psychosis symptoms were measured using the Positive and Negative Syndrome Scale (PANSS) (29). The Marder factor analysis was used to calculate positive, negative, and general psychopathology subscores (30). As a supplemental analysis, we subclassified negative symptoms according to a 2-factor model consisting of expressive (blunted affect, poor rapport, lack of spontaneity in conversation, motor retardation) and experiential (emotional withdrawal, passive social avoidance, active social avoidance) factors (31). We also calculated a PANSS motor subscore, which captures hypokinetic function that may overlap with motor disturbances observed in negative symptoms (32). Depressive symptom severity was assessed using the Montgomery–Åsberg Depression Rating Scale (MADRS) (33) (Supplement).

Current cognitive ability was assessed using the Screen for Cognitive Impairment in Psychiatry (SCIP) (34). The SCIP includes measures of verbal memory (immediate and delayed), working memory, verbal fluency, and processing speed and is a reliable and validated measure of cognitive impairment in psychotic disorders (34,35). Importantly, predictive coding has been linked to cognitive processes such as language and working memory (36). SCIP subtest raw scores were converted to *z* scores using normative data and averaged to create a composite *z* score.

### MRI Acquisition

Neuroimaging data were collected on one of the 2 identical 3T Philips Intera Achieva MRI scanners (Philips Healthcare) located at the Vanderbilt University Institute of Imaging Science. Briefly, a 7- or 10-minute echo-planar imaging resting-state fMRI scan and T1-weighted anatomical scan (1-mm isotropic resolution) were collected for each participant (see the Supplement for scan parameters). Scans were not matched for length.

### MRI Data Processing

Anatomical images were segmented into gray matter, white matter, and cerebrospinal fluid (CSF) with CAT12 (version 12.5; http://www.neuro.uni-jena.de/cat/). Resting-state scans were preprocessed in SPM12 and were 1) realigned to a mean scan, 2) coregistered with the native space structural scan, and 3) spatially normalized to Montreal Neurological Institute (MNI) space using the parameters obtained from the gray matter segmentation normalization. Next, they underwent resting-state denoising procedures: bandpass filtering (0.01–0.1 Hz); regression of CSF, gray matter, and white matter signal; and regression of 12 motion parameters (6 translation and rotation parameters and their first derivative). All resting-state scans went through a quality assurance procedure that included calculating framewise displacement (FD) and temporal signal-to-noise ratio (tSNR). Scans with a mean FD > 0.5 (*n* = 36) or a tSNR lower than the fifth percentile of the distribution of the entire sample (*n* = 32) were excluded from further analysis (37). To ensure adequate coverage of the cerebellum, we excluded scans with <50% coverage of our cerebellar cluster (*n* = 33). After quality control, there were a total of 260 scans for analysis. Included scans had a median coverage of 91.88% of the cerebellar cluster (range 51.88–99.80) (see the Supplement).

### Functional Connectivity Analysis

We calculated cerebellar-DLPFC connectivity by extracting the time course of the blood oxygen level–dependent (BOLD) signal from a mask of the entire cerebellar region identified in Brady *et al.* (20) (Figure S1). Using SPM12 (http://www.fil.ion.ucl.ac.uk/spm) we regressed the *z*-transformed Pearson’s correlation coefficient connectivity maps against the PANSS negative symptom subscore, using age, sex, and scanner as covariates, and an Automated Anatomical Labeling (AAL) bilateral middle frontal gyrus explicit mask in SPM to generate spatial maps of how connectivity from the cerebellar region to the DLPFC varied inversely with negative symptom severity at a voxelwise threshold of *p* < .01. To determine clusterwise significance, we used 3dClustSim in AFNI using a 1-sided test, NN = 3, and the AAL middle frontal gyrus mask, which determined that a cluster *k* > 27 voxels would reach a threshold of *p* < .01. We also performed an unrestricted, brainwide analysis (Supplemental Methods and Supplemental Results). To visualize and quantify the relationship between cerebellar-DLPFC connectivity with behavioral variables, in a post hoc analysis, we calculated region-to-seed connectivity by measuring BOLD correlation between the Brady *et al.* (20) cerebellar region and a 6-mm sphere (seed) placed at the location of maximal connectivity–negative symptom association that we observed in the left DLPFC (MNI x = −26, y = +32, z = +38) using MATLAB (version 2024b Update 5; The MathWorks, Inc.). Then, we correlated connectivity between the cerebellar region and the seed placed at the location of maximal connectivity–negative symptom association in the left DLPFC with PANSS negative symptom subscores.

### Statistical Analysis

Pearson’s correlation coefficients were used to determine the relationships between functional connectivity and behavioral measures. *t* Tests were used to compare continuous outcomes based on dichotomous variables. Analyses of variance were used to compare continuous outcomes based on 3 or more groups. All analyses were conducted in RStudio (version 2023.03.1+446) using alpha < 0.05, except as indicated in the results below to correct for multiple comparisons.

Using the *mediation* package in R, we conducted an indirect effects analysis to test whether there was an indirect effect of cognitive performance on the relationship between negative symptoms and cerebellar-DLPFC connectivity. Indirect effects analysis was conducted using nonparametric bootstrap confidence intervals based on the percentile method and with 1000 simulations. In the models, which consisted of 240 participants, the independent variable was negative symptoms, the indirect effect was the cognitive performance measure, and the dependent variable was cerebellar-DLPFC connectivity. We also conducted sensitivity analysis using the *medsens* package and used the RESI package (https://cran.r-project.org/web/packages/RESI/RESI.pdf) to report effect sizes.

## RESULTS

### Cerebellar-DLPFC Connectivity and Negative Symptoms

#### Association with Negative Symptom Severity Across the Psychosis Spectrum.

**Figure 1.**
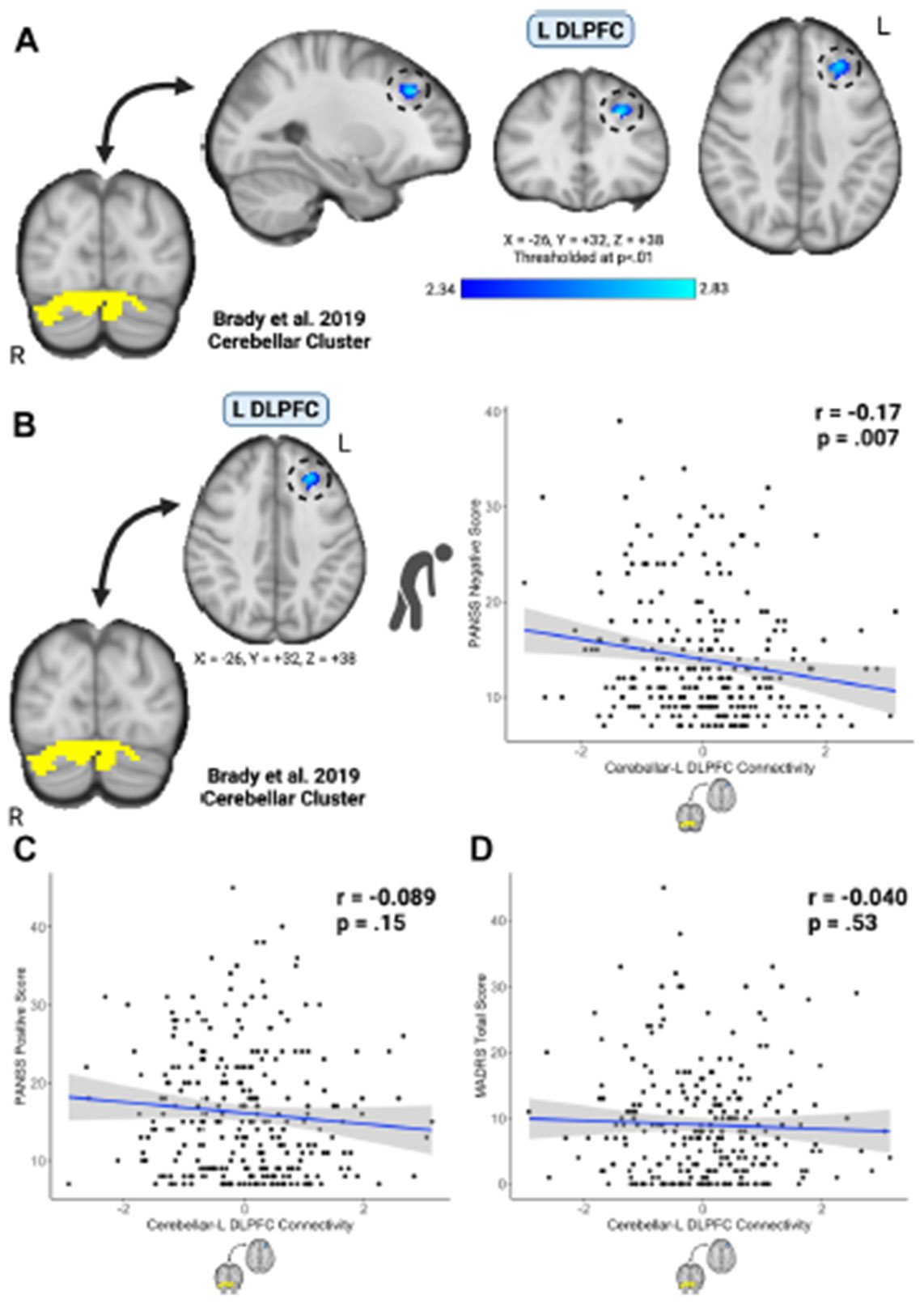
**(A, B)** Cerebellar–dorsolateral prefrontal cortex (DLPFC) connectivity was associated with negative symptom severity across the psychosis spectrum. We observed that higher cerebellar-DLPFC connectivity was associated with lower negative symptom severity (*r* = −0.17, *p* = .007). This connectivity relationship was specific to negative symptoms, as cerebellar-DLPFC connectivity was not associated with **(C)** Positive and Negative Syndrome Scale (PANSS) positive symptoms (*r* = −0.089, *p* = .15) **(D)** or Montgomery–Åsberg Depression Rating Scale (MADRS) depressive symptoms (*r* = −0.040, *p* = .53). L, left; R, right.

When we used the cerebellar region from Brady *et al.* (20) as a seed and regressed connectivity to bilateral DLPFC regions against negative symptom severity, we observed a significant cluster *k* = 111 centered at MNI x = −26, y = +32, z = +38 (*p* < .01). Using the cerebellar region and peak left DLPFC cluster coordinates as seeds, we observed that higher cerebellar-DLPFC connectivity was associated with lower negative symptom severity (*r* = −0.17, *p* = .007, Cohen’s *d* = −0.35) (Figure 1A, B). This connectivity relationship was specific to negative symptoms, as cerebellar-DLPFC connectivity was not associated with PANSS positive symptoms (*r* = −0.089, *p* = .15) (Figure 1C) or MADRS depressive symptoms (*r* = −0.040, *p* = .53) (Figure 1D). In an unrestricted, brainwide analysis, we identified the same left DLPFC cluster as well as clusters in the right posterior cerebellum and brainstem (Supplemental Results and Figure S3).

**Figure 2.**
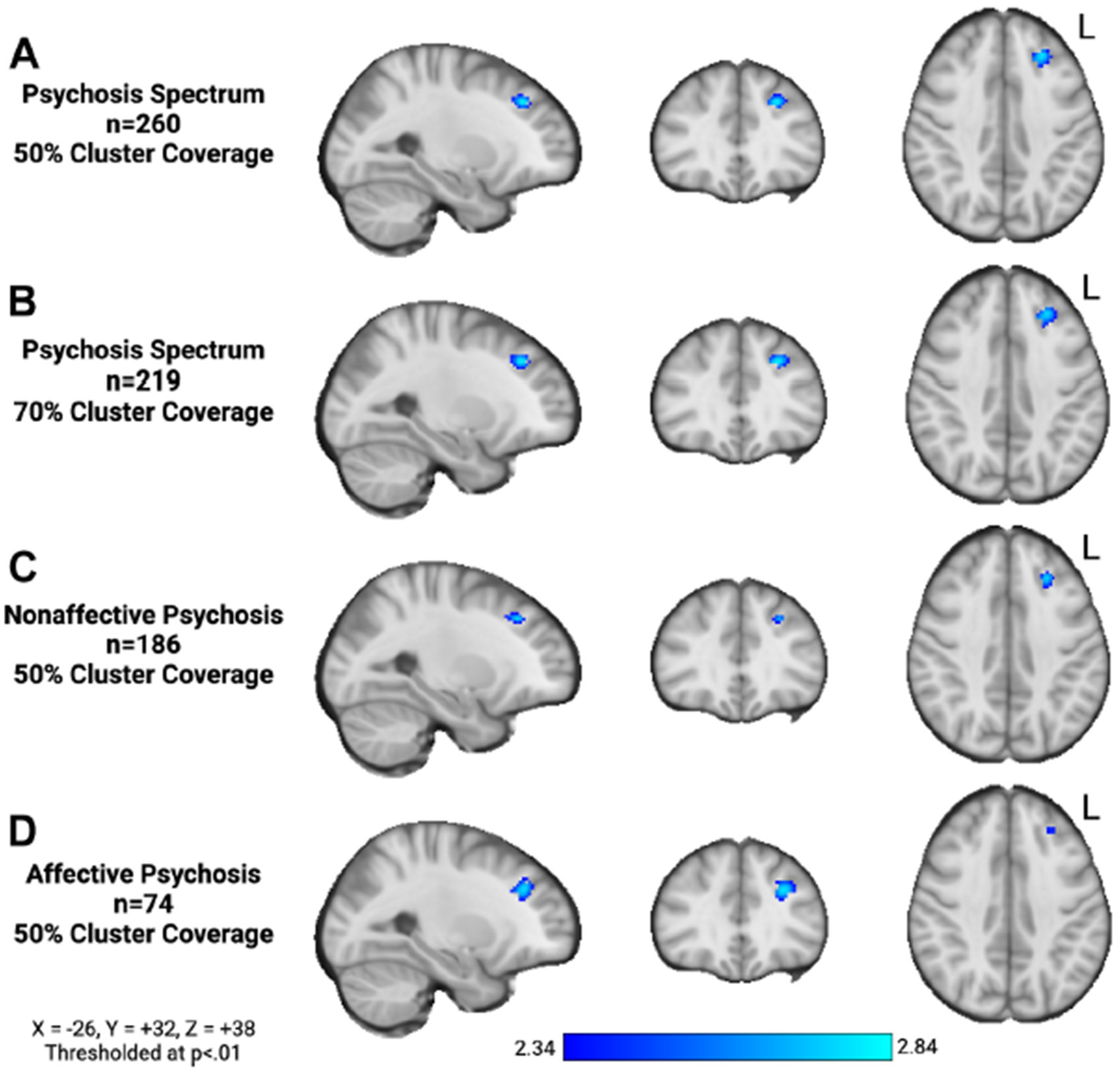
Sensitivity analyses: Cerebellar–dorsolateral prefrontal cortex (DLPFC) connectivity is associated with negative symptom severity across the psychosis spectrum. **(A, B)** To confirm that our result was not affected by scanner coverage of our cerebellar region, we repeated our neuroimaging analyses for scans with >70% coverage of the cerebellar region (*n* = 219) and observed similar results such that higher cerebellar-DLPFC connectivity was associated with lower negative symptom severity (*r* = −0.18, *p* = .008). To ensure that our result was not specific to nonaffective psychosis, we repeated our analysis in psychosis subgroups: **(C)** nonaffective psychosis (*n* = 186, *r* = −0.18, *p* = .016) and **(D)** affective psychosis (*n* = 74, *r* = −0.26, *p* = .025). In all subgroup analyses, we observed similar relationships between cerebellar-DLPFC connectivity and negative symptom severity. L, left.

To confirm that our result was not affected by scanner coverage of our cerebellar region, we repeated our neuroimaging analyses for scans with >70% coverage of the cerebellar region (*n* = 223) and observed similar results such that higher cerebellar-DLPFC connectivity was associated with lower negative symptom severity (*r* = −0.18, *p* = .008, *d* = −0.37) (Figure 2A, B). Percent cluster coverage was not associated with cerebellar-DLPFC connectivity (*r* = 0.021, *p* = .73) or negative symptom severity (*r* = −0.073, *p* = .25).

#### Psychosis Subtypes.

**Figure 3.**
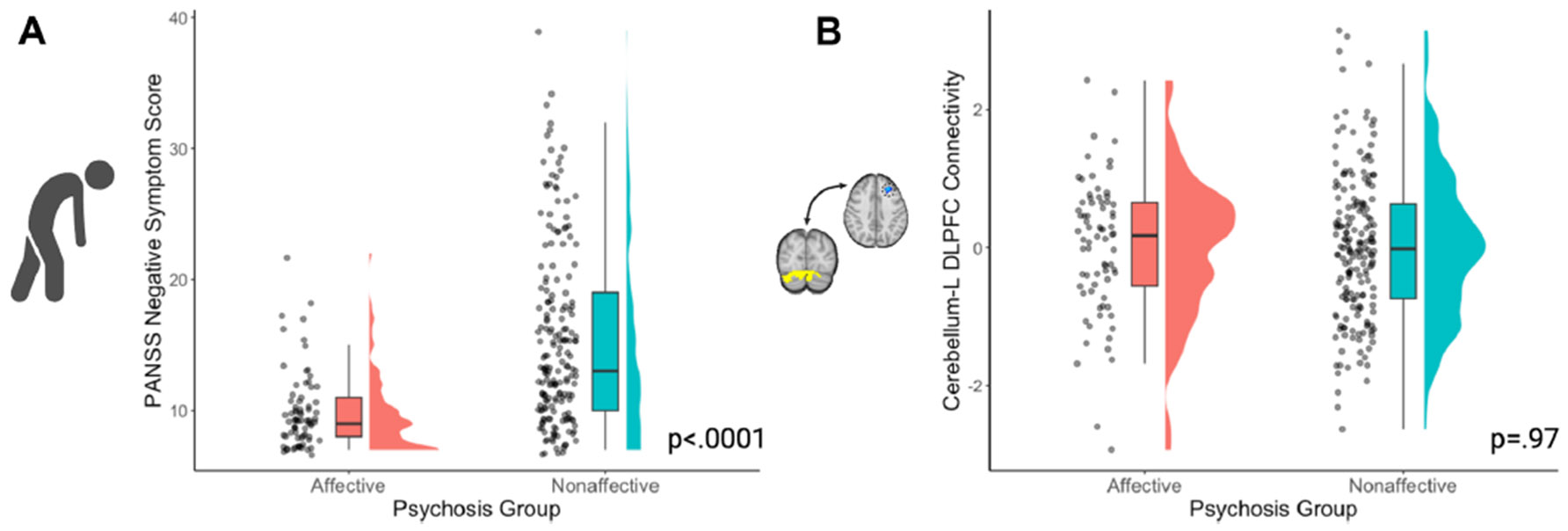
Negative symptom severity, but not cerebellar–dorsolateral prefrontal cortex (DLPFC) connectivity, was associated with psychosis subtype. **(A)** In a large sample of individuals with psychosis spectrum disorders (*N* = 260), Positive and Negative Syndrome Scale (PANSS) negative symptom severity was associated with psychosis subtype (*t*_256.33_ = −9.1723, *p* < 2.2 × 10^−16^). **(B)** We calculated connectivity between the previously identified cerebellar region from Brady *et al.* (20) and the left (L) DLFPC region that we identified in our analyses. Cerebellar-DLPFC connectivity did not differ by psychosis type (*t*_144.99_ = 0.0415, *p* = .967).

In our psychosis spectrum disorder sample (*N* = 260), there were associations between PANSS negative symptom severity and psychosis subtype (i.e., affective vs. nonaffective) (*t*_256.33_ = −9.1723, *p* < 2.2 × 10^−16^) (Figure 3A) and psychotic disorder diagnosis (*F*_6_ = 10.902, *p* = 8.54 × 10^−11^) (Figure S2). Cerebellar-DLPFC connectivity was not associated with psychosis subtype (*t*_144.99_ = 0.0415, *p* = .967) (Figure 3B).

To ensure that our result was not specific to nonaffective psychosis, we repeated our analysis in psychosis subgroups: nonaffective psychosis (*n* = 186, *r* = −0.18, *p* = .016) (Figure 2C) and affective psychosis (*n* = 74, *r* = −0.26, *p* = .025) (Figure 2D). In all subgroup analyses, we observed a single significant cluster in the left DLPFC and significant relationships between cerebellar-DLPFC connectivity and negative symptom severity.

In a general linear model predicting negative symptom severity that included cerebellar-DLPFC connectivity, psychosis subtype (affective vs. nonaffective psychosis), age, and sex as predictors, cerebellar-DLPFC connectivity, nonaffective psychosis, and age were significantly related to negative symptom severity (*F*_4,255_ = 15.31, *p* = 3.077 × 10^−11^; cerebellar-DLPFC connectivity *t* = −2.918, *p* = .00383; nonaffective psychosis *t* = 6.447, *p* = 5.66 × 10^−10^; age *t* = −2.032, *p* = .043).

#### Psychomotor Disturbances.

Given the cerebellum’s prominent role in motor function and the psychomotor disturbances that comprise negative symptoms, we also tested whether connectivity was related to a PANSS motor subscore. Higher cerebellar-DLPFC connectivity was associated with lower motor symptom severity (*r* = −0.14, *p* = .023, *d* = −0.28).

Results were not affected by antipsychotic medication, duration of illness, negative symptom domains, or head motion (Supplement).

### Cerebellar-DLPFC Connectivity and Cognitive Function

#### Association with Delayed Verbal Learning.

**Figure 4.**
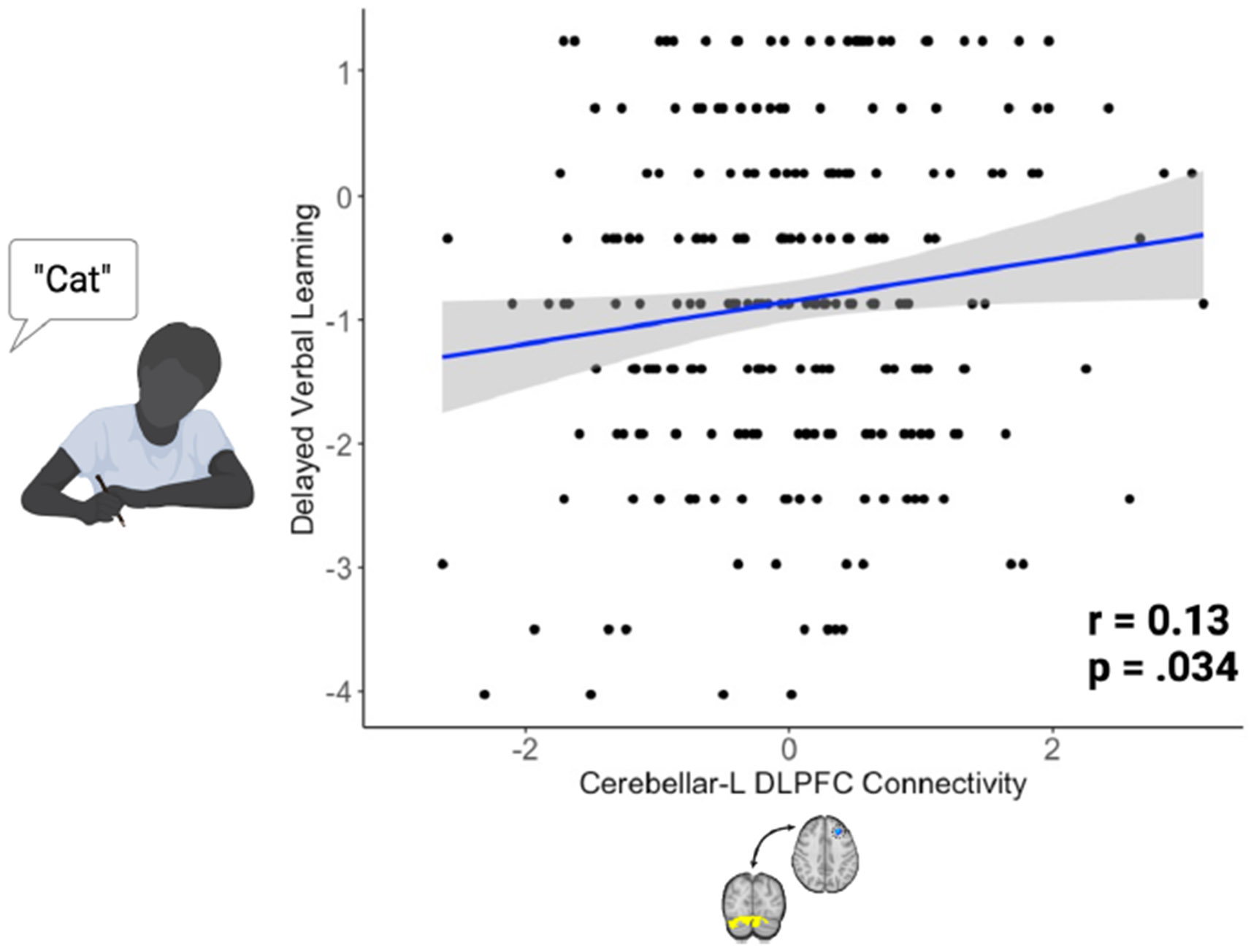
Cerebellar–dorsolateral prefrontal cortex (DLPFC) connectivity was associated with delayed verbal learning. Given existing evidence supporting the role of both the cerebellum and DLPFC in cognitive performance, we investigated the relationships between cerebellar-DLPFC connectivity and cognitive performance. Higher cerebellar-DLPFC connectivity was associated with better delayed verbal learning (*r* = 0.13, *p* = .034). Connectivity was not related to cognitive performance in any other domains. L, left.

Given existing evidence supporting the role of both the cerebellum and DLPFC in cognitive performance, we investigated the relationships between cerebellar-DLPFC connectivity and cognitive performance. Cerebellar-DLPFC connectivity was not associated with SCIP total score (*r* = 0.084, *p* = .18). In post hoc analyses, higher cerebellar-DLPFC connectivity was associated with better delayed verbal learning (*r* = 0.13, *p* = .034, *d* = 0.26) (Figure 4) but was not associated with any other cognitive domains on the SCIP.

#### Negative Symptoms Are Broadly Associated with Cognitive Performance.

PANSS negative symptom severity was associated with SCIP total score (*r* = −0.38, *p* = 4.58 × 10^−10^, *d* = −0.82). Then we, tested whether this association was specific to cognitive domains. Negative symptom severity was associated with all 5 cognitive domains on the SCIP (Bonferroni-corrected *p* = .05/5 = .01): verbal learning-immediate (*r* = −0.31, *p* = 4.632 × 10^−7^, *d* = −0.65), working memory (*r* = −0.34, *p* = 2.063 × 10^−8^, *d* = −0.72), verbal fluency (*r* = −0.17, *p* = .0053, *d* = −0.35), verbal learning-delayed (*r* = −0.27, *p* = 1.19 × 10^−5^, *d* = −0.56), and processing speed (*r* = −0.25, *p* = 4.75 × 10^−5^, *d* = −0.52).

#### Delayed Verbal Learning Affects the Relationship Between Negative Symptom Severity and Cerebellar-DLPFC Connectivity.

**Figure 5.**
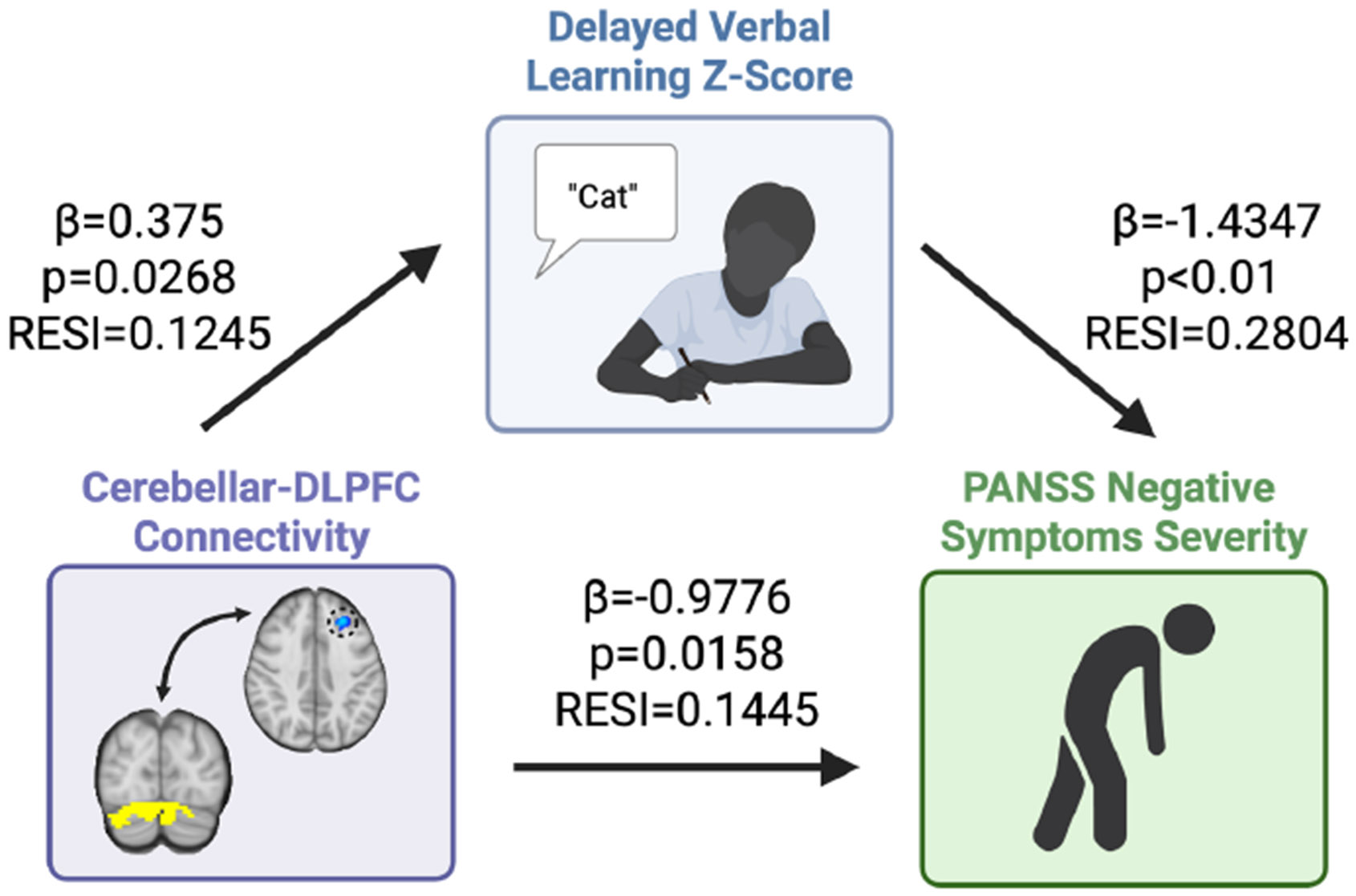
Delayed verbal learning affected the relationship between cerebellar–dorsolateral prefrontal cortex (DLPFC) connectivity and negative symptoms. Given the relationships between cerebellar-DLPFC connectivity and both negative symptom severity and delayed verbal learning, we performed an indirect effects analysis to determine whether delayed verbal learning indirectly affected the relationship between cerebellar-DLPFC connectivity and negative symptoms. Approximately 25.44% of the total effect of cerebellar-DLPFC connectivity on negative symptoms was affected by delayed verbal learning performance. We reported beta values and effect size estimates using RESI. PANSS, Positive and Negative Syndrome Scale.

We performed exploratory indirect effects analyses to determine whether SCIP total score, verbal memory (immediate and delayed), working memory, verbal fluency, or processing speed indirectly affected the relationship between cerebellar-DLPFC connectivity and cognitive symptoms (Table S3). Only delayed verbal learning had a significant indirect effect on the relationship between cerebellar-DLPFC connectivity and negative symptom severity (Figure 5). In this model, the average causal mediation effect (ACME) of the delayed verbal learning *z* score (ACME = −0.2487 [−0.5270 to −0.030]) was significant (*p* = .026), suggesting that delayed verbal learning accounts for a portion of the effect of cerebellar-DLPFC connectivity on negative symptoms. The proportion of the relationship affected by the delayed verbal learning *z* score was 0.2544 ([0.0299 to 0.78], *p* = .040), indicating that approximately 25.44% of the total effect of cerebellar-DLPFC connectivity on negative symptoms is affected by delayed verbal learning performance. The average direct effect (ADE) of cerebellar-DLPFC connectivity on negative symptoms (ADE = −0.7289 [−1.4606 to −0.040]) and the total effect of cerebellar-DLPFC connectivity on negative symptoms (estimate = −0.9776 [−1.7733 to −0.270]) were also significant (*p* = .046 and *p* = .014, respectively).

These results suggest that there is a significant total and direct effect of cerebellar-DLPFC connectivity on negative symptoms. Additionally, the model suggests that there is an indirect effect of the delayed verbal learning *z* score on the relationship between cerebellar-DLPFC connectivity and negative symptom severity.

A sensitivity analysis was conducted to determine how sensitive the ACME was to violations of the sequential ignorability assumption. The analysis suggested that the indirect effect was relatively robust to moderate violations of the ignorability assumption. The estimated correlation between the error terms of the latent linear models for the indirect effect and outcome models was −0.30. The proportion of variance in the indirect effect and outcome that would need to be explained by an unmeasured confounder to invalidate the indirect effect was 0.09, and the proportion of the total variance that would need to be explained by an unmeasured confounder to invalidate the indirect effect was 0.0794; both were small.

## DISCUSSION

The current work links cerebellar-DLPFC connectivity to negative symptom severity in schizophrenia by investigating connectivity patterns in a large psychosis spectrum sample. Consistent with previous work (20), we observed that higher cerebellar-DLPFC connectivity was associated with lower negative symptom severity. Our work extends the relationship between cerebellar-DLPFC connectivity and negative symptoms into a 5-fold larger sample of psychosis spectrum disorders that was recruited in an entirely different geographic location. We showed that the relationship between connectivity and negative symptom severity is specific to negative symptoms and robust across psychosis subtypes (i.e., nonaffective vs. affective psychosis), negative symptom subdomains, and a broad duration of illness.

These findings suggest that cerebellar-DLPFC connectivity is a promising target for intervention (e.g., pharmacologic, psychotherapeutic, neuromodulatory) because of its specificity to negative symptom severity versus other psychosis symptom domains and persistence across all phases of illness and psychosis spectrum diagnoses. Interestingly, our exploratory brainwide analysis did not identify alternative regions in the frontal or somatomotor cortices.

Consistent with previous literature, we found relationships between negative symptom severity and cognitive performance that were not domain specific. Various work has linked negative symptom pathology to deficits in cognitive processes, such as reward sensitivity and processing (19) and predictive coding, which broadly contribute to motor function, language, withdrawal, and affect. Importantly, the cerebellum has been implicated as one potential neural system that drives these fundamental processes in both healthy and psychosis populations (22-25). One mechanism by which this may occur is through changes in the Purkinje cell function, which has been identified in psychosis. Purkinje cells operate as a constant moderator of hundreds of thousands of sensory-based inputs within the cerebellar structure, facilitating complex computations that are then output by the deep nuclei of cerebellum to the cerebrum (17,38). Among individuals with psychosis, these cells may operate more erroneously, impeding this delicate computation process and the accurate predictions that underlie fundamental processes for behaviors such as language, social interaction, movement, and affect (39).

In support, previous work has shown that cognitive performance mediated the relationship between cerebellum and psychosis symptoms (24), including recent work by Cao *et al.* (40), who also observed a mediating effect of verbal ability and then showed that cerebellar rTMS improved cognitive performance. When we tested whether cognitive performance affected the relationship between cerebellar-DLPFC connectivity and negative symptoms, we observed an indirect effect of delayed verbal learning, but no other cognitive domains, on this connectivity–negative symptom relationship. These novel findings extend previous work connecting negative symptoms, verbal learning, and DLPFC connectivity. A recent study found that negative symptoms were associated with verbal learning but not with other measures of cognition in individuals with early-onset psychosis (41), and delayed verbal memory distinguishes those at high risk for schizophrenia from healthy control individuals (42). Another study found a link between DLPFC connectivity, verbal learning and memory, and negative symptoms in schizophrenia (43).

Addressing both cognitive and negative symptoms of schizophrenia is becoming increasingly important, and our indirect effects analysis provides further evidence that cerebellar connectivity may be a pathway through which delayed verbal learning performance and negative symptoms can be targeted (40,44). Importantly, the cerebellum has also been linked to psychomotor disturbance and cognitive performance in individuals at clinical high risk for psychosis, suggesting a role of the cerebellum across illness phases (45-48), increasing the therapeutic potential of this brain target.

Our findings provide additional evidence supporting the role of the cerebellum in cognitive performance. The cerebellum is the most neuron-dense region of the brain, containing roughly 80% of the brain’s neurons (49). While the cerebellum has been most widely associated with balance and coordination, recent research has increasingly demonstrated the cerebellum’s role in cognitive performance. The cerebellar region that we studied has been linked to social, linguistic, and spatial function in a recent precision functional mapping analysis (50), consistent with our findings relating connectivity from this region to negative symptoms and verbal learning. The cerebellum has been identified as a critical node within a subcortical network that determines the efficiency with which verbal input is processed (51), which is also prominently involved in the procedural memory system (52). Verbal working memory plays a substantial role in an individual’s verbal learning abilities via disruption within internal models that support articulation (53) and interference in phonological encoding, verbal rehearsal, and retrieval (54).

The novel relationships that we identified between cerebellar connectivity and negative symptoms provide additional evidence that the cerebellum may be a central driver of multiple domains of psychiatric symptoms, including psychomotor disturbance and cognitive performance. In our analyses, cerebellar-DLPFC connectivity was also related to a motor symptom subscore. Another recent analysis by our group linked cerebellar connectivity to the cognitive and motor components of psychomotor performance (25). Negative symptoms are a heterogeneous construct consisting of decreased speech production, amotivation, anhedonia, and asociality. Therefore, it is likely that the cerebellum has a common role in negative symptoms and psychomotor function. This is frequently observed in negative symptoms, specifically in the presentation of alogia, which has been linked to hypoconnectivity between the cerebellum and somatosensory networks (55). While our findings indicate dysconnectivity between the cerebellum and DLPFC cortex, rather than the somatosensory network, the cognitive dysfunction findings suggest a persistent critical need for continued examination of the cerebellum’s role in cognitive functioning in psychosis, especially in the context of verbal memory and verbal learning.

Our results also provide further support for cerebellar neuromodulation interventions. Although small, the cerebellar rTMS study from Brady *et al.* (20) demonstrated that increasing cerebellar-DLPFC connectivity led to reductions in negative symptom severity. Given that this network edge has nodes in the cerebellum and DLPFC, it is possible that rTMS interventions could use either region as a target. Our analysis supports the bidirectional nature of this relationship. Our connectivity analysis used the cerebellar region as a seed, whereas Brady *et al.* (20) seeded the DLPFC region, and our results showed reproducible relationships between cerebellar-DLPFC connectivity and negative symptom severity. However, given the neuronal density of the cerebellum and recent work showing dramatic individual differences in network topography of the PFC (56), the cerebellum may be a preferable rTMS target. Cerebellar rTMS has been extensively studied in neurological and motor disorders (57-59) and is now being increasingly studied in schizophrenia (40,60,61).

Strengths of our study include the use of a large sample of individuals with psychosis spectrum disorders across all phases of illness, including both early and chronic illness. Participants were taking a range of antipsychotic medications, which were not associated with negative symptoms or cerebellar-DLPFC connectivity. Finally, our sensitivity analyses ensured that the relationships that we observed between connectivity and negative symptoms were valid across psychosis diagnoses, including both affective and nonaffective psychoses. These findings suggest a target for intervention for negative symptoms across the psychosis spectrum, a significant implication given the lack of any U.S. Food and Drug Administration–approved medications for negative symptoms. Another strength of our analysis was the inclusion of cognitive assessment data in indirect effects analyses. Although causality cannot be determined from an indirect effects analysis using cross-sectional data, our results are consistent with recent findings (40) and suggest that future interventional studies should assess negative symptoms and cognitive performance to better understand their relationship.

There are several limitations of our analyses. Our study included cross-sectional data collected from one site, although in an entirely different geographic location than Brady *et al.* (20). Neuroimaging scan sequences were not optimized for the cerebellum, and as a result, the minimum cerebellar region of interest coverage was 50% to 70%. Additionally, while we made reliable assessments using the PANSS and the SCIP to measure symptoms, these scales are general assessments of negative symptoms and cognitive performance and therefore limit the ability to precisely differentiate specific symptoms and cognitive domains as heterogeneous constructs. More nuanced assessment of negative symptom domains [e.g., Clinical Assessment Interview for Negative Symptoms (62), Brief Negative Symptoms Scale (63)], behavioral measures of negative symptoms (e.g., anhedonia), and more sophisticated cognitive tasks are likely to yield stronger brain-behavior relationships. As a result, interpretations of our results in relation to underlying mechanisms are limited.

### Conclusions

We used data from a large sample of individuals with psychosis spectrum disorders to provide novel evidence supporting the association between cerebellar-DLPFC connectivity and negative symptom severity and delayed verbal learning. These results should be tested in a larger, randomized, sham-controlled trial of cerebellar rTMS in people with psychotic disorders.

## Supplementary Material

Supplementary Information

Supplementary material cited in this article is available online at https://doi.org/10.1016/j.bpsc.2025.07.013.
