# Supplementary material for: Cerebellar-Prefrontal Connectivity Predicts Negative Symptom Severity Across the Psychosis Spectrum
Source: Biol Psychiatry Cogn Neurosci Neuroimaging. Author manuscript; Available in PMC 2026 Feb 1. (PMC12861324; doi:10.1016/j.bpsc.2025.07.013)
Supplement: Supplementary Information [file NIHMS2139748-supplement-Supplementary_Information.pdf]

SUPPLEMENTARY INFORMATION

Cerebellar-Prefrontal Connectivity Predicts Negative Symptom Severity Across the Psychosis Spectrum

Yarrell *et al.*

Supplemental Materials

Supplemental Methods

Participants

Data came from a repository of 361 individuals with psychotic disorders with complete neuroimaging and behavioral data who participated in one of three neuroimaging projects conducted in the Department of Psychiatry and Behavioral Sciences at Vanderbilt University Medical Center (VUMC, CT00762866; 1R01MH070560; 1R01MH102266). Participants were recruited from the Psychotic Disorders Program at VUMC. All studies were approved by the Vanderbilt Institutional Review Board and all individuals provided written informed consent prior to participating in the studies. The Structured Clinical Interview for DSM-IV or DSM-5 (SCID-IV, SCID-5) was administered to all study participants to confirm diagnoses (1, 2). Exclusion criteria were similar across the three studies, including age under 16/18 years or over 65 years (55 years in 1R01MH102266); estimated premorbid IQ less than 70 based on the Wechsler Test of Adult Reading; history of significant head trauma, medical illness, or central nervous system disorder; pregnancy or lactation; substance abuse within the last 1 month for patients (3 months in 1R01MH102266); and MRI contraindicators.

Assessments

Psychosis symptoms were measured using the Positive and Negative Syndrome Scale (PANSS) (3), a clinician-rated assessment of positive, negative, and general psychopathology symptoms over the past two weeks. The Marder factor analysis was used to calculate positive, negative, and general psychopathology subscores (4). The Marder factor of negative symptoms, comprises 7 items of the PANSS: blunted affect, emotional withdrawal, poor rapport, passive/apathetic withdrawal, motor retardation, active social avoidance, and lack of spontaneity in conversation. We also subclassified negative symptoms according to a two-factor model consisting of expressive (blunted affect, poor rapport, lack of spontaneity in conversation, motor retardation) and experiential factors (emotional withdrawal, passive social avoidance, active social avoidance) (5). We also calculated a PANSS motor subscore, established in the literature to capture hypokinetic function which may overlap significantly with motor disturbances observed in negative symptoms. This score was calculated by summing the scores for mannerisms and posturing; motor retardation;

and disturbance of volition (6). Depressive symptom severity was assessed using the Montgomery–Åsberg Depression Rating Scale (7).

MRI Acquisition

Scan parameters are shown in table below.

Scan Types	TR (msec)	TE (msec)	Flip Angle	Voxel Size (mm^3)	Volumes	FOV mm (x)	Slice Thickness (mm)
T1	8	4	5	1	1	170	1
	9	5	8	1	1	170	1
Resting-State fMRI	2000	35	79	3	203	240	4
	2000	28	90	3	210	240	3.2
	2000	28	90	3	203	240	3.2
	2000	25	90	3	300	240	3

Unrestricted, Brain-wide Cerebellar Functional Connectivity Analysis

We calculated cerebellar-DLPFC connectivity by extracting the time course of the blood-oxygen-level-dependent (BOLD) signal from a mask of the entire cerebellar region identified in Brady et al. (8) (displayed on cerebellar flatmap in Supplemental Figure 1). Using SPM12 (SPM – Statistical Parametric Mapping, http://www.fil.ion.ucl.ac.uk/spm) we regressed the z-transformed Pearson's correlation coefficient connectivity maps against the PANSS negative symptom subscore, using age, sex, and scanner as covariates to generate spatial maps of how brain-wide connectivity from the cerebellar region inversely varied with negative symptom severity at a voxelwise threshold of $p<.01$.

Supplemental Results

In Unrestricted, Brain-wide Analysis, Cerebellar-DLPFC Connectivity is Associated with Negative Symptom Severity Across the Psychosis Spectrum

When we used the cerebellar region from Brady et al., 2019 as a seed and regressed brain-wide connectivity against negative symptom severity, controlling for age, sex, and scanner, we observed a significant cluster k = 131, centered at MNI X = -26, Y = +32, Z = +38 in the left DLPFC (Supplemental Figure 3). There were two other larger clusters identified in the right posterior cerebellum (k = 1006 at MNI X = +28, Y = -76, Z = -22) and brainstem (k=607 at MNI X = +14, Y = -28, Z = -56).

Antipsychotic Medication Dose is Not Associated with Negative Symptom Severity or Cerebellar-DLPFC Connectivity

Confirming our results were not affected by antipsychotic medication, chlorpromazine equivalents were not associated with negative symptom severity ($r=0.034$, $p=.63$) or cerebellar-DLPFC connectivity ($r= -0.062$, $p=.37$).

Duration of Illness Does Not Predict Negative Symptom Severity or Cerebellar-DLPFC Connectivity

Because our psychosis spectrum sample included individuals across the lifespan, ranging from a first episode of psychosis to chronic illness, we then tested if the relationship between negative symptoms and connectivity differed by duration of illness. In our sample, participants had a median illness duration of 17.00 months (mean 75.74 months, range 0.0 to 567.0 months). Duration of illness was not associated with cerebellar-DLPFC connectivity ($r=-0.049$, $p=.43$) or negative symptom severity ($r=-0.12$, $p=.062$).

In a general linear model predicting negative symptom severity based on age, sex, cerebellar-DLPFC connectivity, and duration of illness, only cerebellar-DLPFC connectivity was a significant predictor ($F(5,254)=3.457$, $p=.0049$; cerebellar-DLPFC connectivity $t=-2.693$, $p=.0076$; duration of illness $t=0.005$, $p=1.0$). In a general linear model predicting cerebellar-DLPFC connectivity based on age, sex, negative symptom severity, and duration of illness, only negative symptom severity was a significant predictor ($F(5,254)=2.084$, df1=5, df2=254, $p=.068$; negative symptom severity $t=-2.693$, $p=.0076$; duration of illness $t=-1.39$, $p=.17$).

Cerebellar-DLPFC Connectivity is Associated with Both Expressive and Experiential Negative Symptom Domains

Negative symptoms have previously been subclassified into an expressive factor and an experiential factor, so we performed an exploratory analysis to test if our connectivity-negative symptoms relationship was specific to either factor. We observed significant relationships between cerebellar-DLPFC connectivity and both the expressive factor ($r=-0.17$, $p=.0058$) and the

experiential factor (r=-0.15, p=.016), but these correlations were not significantly different (Fisher's z=0.2455, p=.8060).

Head Motion Does Not Predict Negative Symptom Severity or Cerebellar-DLPFC Connectivity

To confirm our results were not related to head motion, head motion was not associated with negative symptom severity (r=-0.044, p=.48) or cerebellar-DLPFC connectivity (r=0.050, p=.42). We then tested if head motion (framewise displacement) predicted 1) negative symptom severity and 2) cerebellar-DLPFC connectivity.

In a general linear model predicting negative symptom severity based on head motion, cerebellar-DLPFC connectivity, age, and sex, only cerebellar-DLPFC connectivity and age were significant predictors (F(4,255)=4.338, p=.0021; cerebellar-DLPFC connectivity t=-2.708, p=.0072; age t=-2.555, p=.011; head motion t=0.63, p=.53).

In a general linear model predicting cerebellar-DLPFC connectivity based on head motion, negative symptom severity, age, and sex, only negative symptom severity was a significant predictor (F(4,255)=2.096, p=.082; negative symptom severity t=-2.708, p=.0072; head motion t=0.83, p=.41).

In a general linear model predicting cerebellar-DLPFC connectivity based on age, sex, head motion, duration of illness, and negative symptom severity, only negative symptom severity was a significant predictor (F(5,254)=2.084, p=.067; negative symptom severity t=-2.693, p=.0078).

Negative Symptoms, but Not Positive or Depressive Symptoms, Predict Cerebellar-DLPFC Connectivity

To confirm that cerebellar-DLPFC connectivity was only associated with negative symptoms and not positive or depressive symptoms, we performed a general linear model predicting cerebellar-DLPFC connectivity based on age, sex, PANSS negative subscore, PANSS positive subscore, and MADRS total score. In this model, only PANSS negative symptom severity was a significant predictor (F(5,253)=1.563, p=.17; PANSS negative symptom severity t=-2.295, p=.023; PANSS positive symptom severity t=-0.875, p=.38; MADRS total t=-0.090, p=.93).

Scanner Site

Cerebellar-DLPFC connectivity did not differ by scanner (t(164.25)=0.057, p=.95). In a general linear model predicting cerebellar-DLPFC connectivity based on age, sex, head motion, scanner, and negative symptom severity, only negative symptom severity was a significant predictor (Beta = -0.028, t(254)=-2.76, p=.0063).

SCIP Total Z-Score and SCIP Sub-Domains Did Not Affect the Relationship Between Cerebellar-Prefrontal Connectivity and Negative Symptoms

We assessed whether total SCIP z-score, immediate verbal learning test z-score, working memory test z-score, verbal fluency test z-score, or processing speed test z-score indirectly affected the relationship between cerebellar prefrontal connectivity and negative symptoms. All indirect effects analyses included the cerebellar-prefrontal connectivity as the independent variable, negative symptoms as the dependent variable, and the cognitive z-score as the indirect effect. All indirect effects analyses included a sample size of 240 and ran 1000 simulations using the percentile method. For all models, the average direct effect and total effect of cerebellar-prefrontal connectivity on negative symptoms was significant, but none of the average causal mediation estimates or proportion affected by the cognitive z-score were significant (see Supplemental Table 2). For all models, the total effect was -0.978* [-1.785, -0.23], p=0.008.

Supplemental Tables & Figures

Supplemental Table 1. Demographics

Demographic	Psychosis (n=260)
Female, n (%)	87 (33.5)
Age (years), Mean (SD)	28.2 (11.0)
Duration of Illness (years)	
Mean (SD)	6.31 (9.26)
Range	0 - 47.25
Race, n (%)	
White	180 (69.2)
Black	66 (25.4)
Asian	2 (0.77)
Native American	3 (1.6)
Other	9 (3.5)
PANSS, Mean (SD)	
Positive	16.1 (8.1)
Negative	13.9 (6.5)
General	29.7 (8.6)
Total	59.7 (18.4)
MADRS, Mean (SD)	9.0 (8.4)
SCIP Total Z-Score, Mean (SD)	-0.89 (0.98)
Chlorpromazine Equivalents, Mean (SD)	378.8 (576.6)
Diagnosis, n, (%)	
Nonaffective Psychosis	186 (71.5)
Affective Psychosis	74 (28.5)

Supplemental Table 2. Psychosis Spectrum Diagnoses Included in Sample

Diagnosis, n, (%)	Psychosis (n=260)
Schizophrenia	81 (31.2)
Schizophreniform	73 (28.1)
Bipolar Disorder with Psychotic Features	71 (27.3)
Schizoaffective Disorder	28 (10.8)
Major Depressive Disorder with Psychotic Features	3 (1.2)
Brief Psychotic Disorder	3 (1.2)
Other Psychotic Disorder	1 (0.38)

Supplemental Table 3. SCIP Indirect Effects Models of Prefrontal-Cerebellar Connectivity and Negative Symptoms

Indirect Effect	Average Direct Effect	Average Causal Mediation Estimate	Proportion Affected
Total SCIP z-Score	-0.754* [-1.459, -0.08]	-0.224 [-0.559, 0.10]	0.229 [-0.158, 0.71]
Verbal Learning Test z-Score	-0.838* [-1.550, -0.12]	-0.140 [-0.415, 0.16]	0.143 [-0.295, 0.55]
Working Memory Test z-Score	-0.795* [-1.517, -0.10]	-0.183 [-0.461, 0.11]	0.187 [-0.240, 0.66]
Verbal Fluency Test z-Score	-0.9883* [-1.7113, -0.25]	0.0106 [-0.1442, 0.16]	-0.0109 [-0.2594, 0.18]
Processing Speed Test z-Score	-0.9225* [-1.7269, -0.18]	-0.0551 [-0.2702, 0.16]	0.0564 [-0.3290, 0.33]

*Note: *indicates a p-value less than 0.05.*

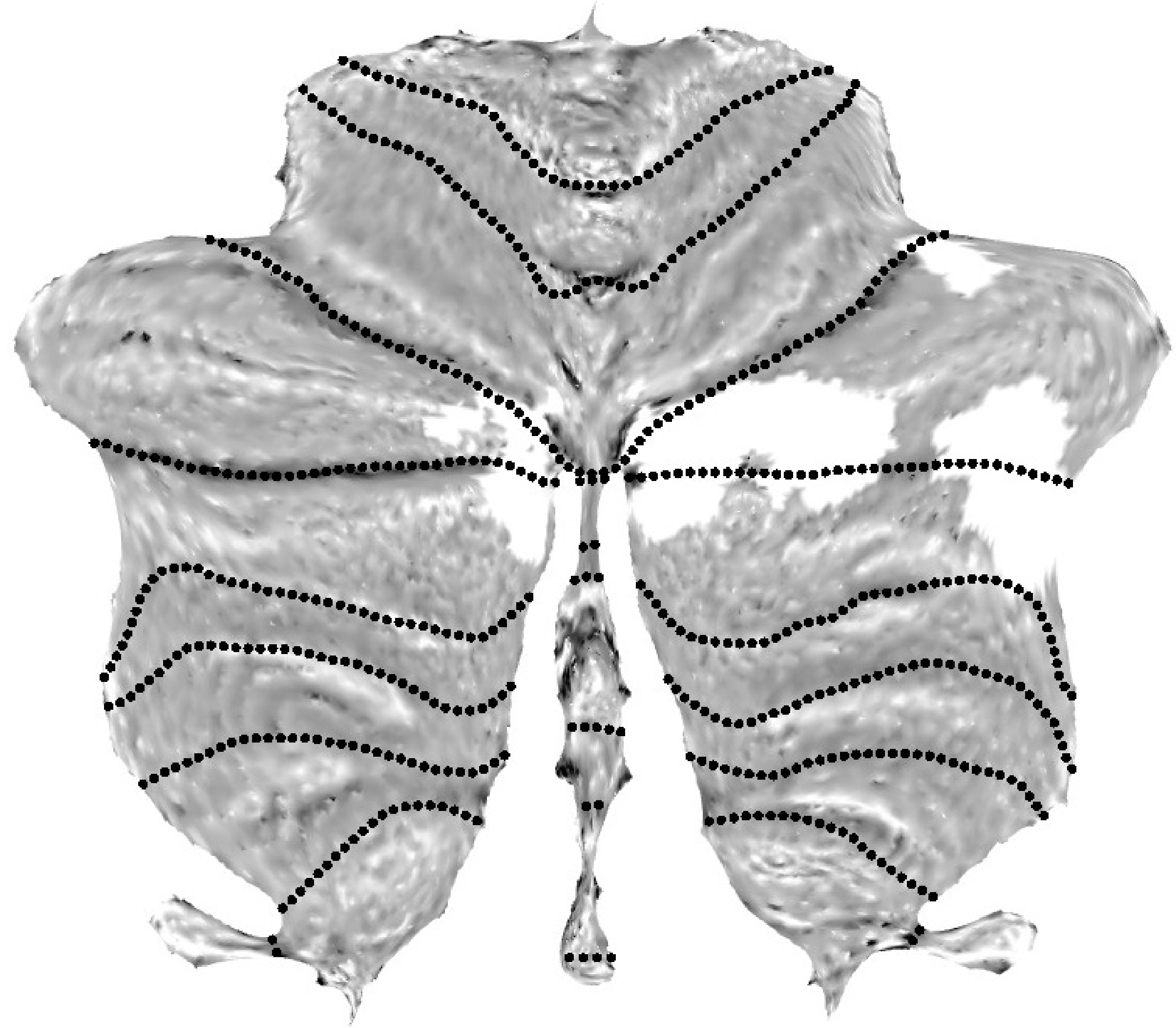

Supplemental Figure 1. Cerebellar Cluster Displayed on Cerebellar Flatmap. We calculated cerebellar-prefrontal connectivity by extracting the time course of the blood-oxygen-level-dependent signal from the cerebellar region identified in Brady et al. (8), displayed here in white on a cerebellar flatmap.

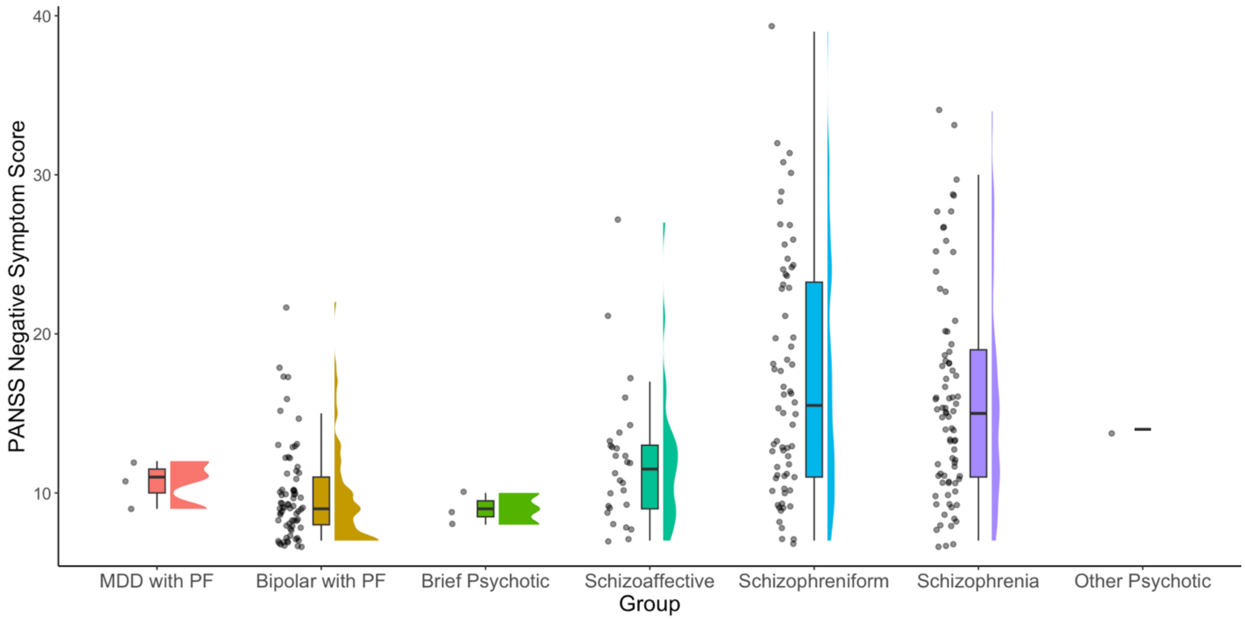

Supplemental Figure 2. Negative Symptom Severity is Associated with Psychotic Disorder Diagnosis. In a large sample of individuals with psychosis spectrum disorders (n=260), PANSS negative symptom severity was associated with psychotic disorder diagnosis (F(6)=10.902, p=8.54e-11).

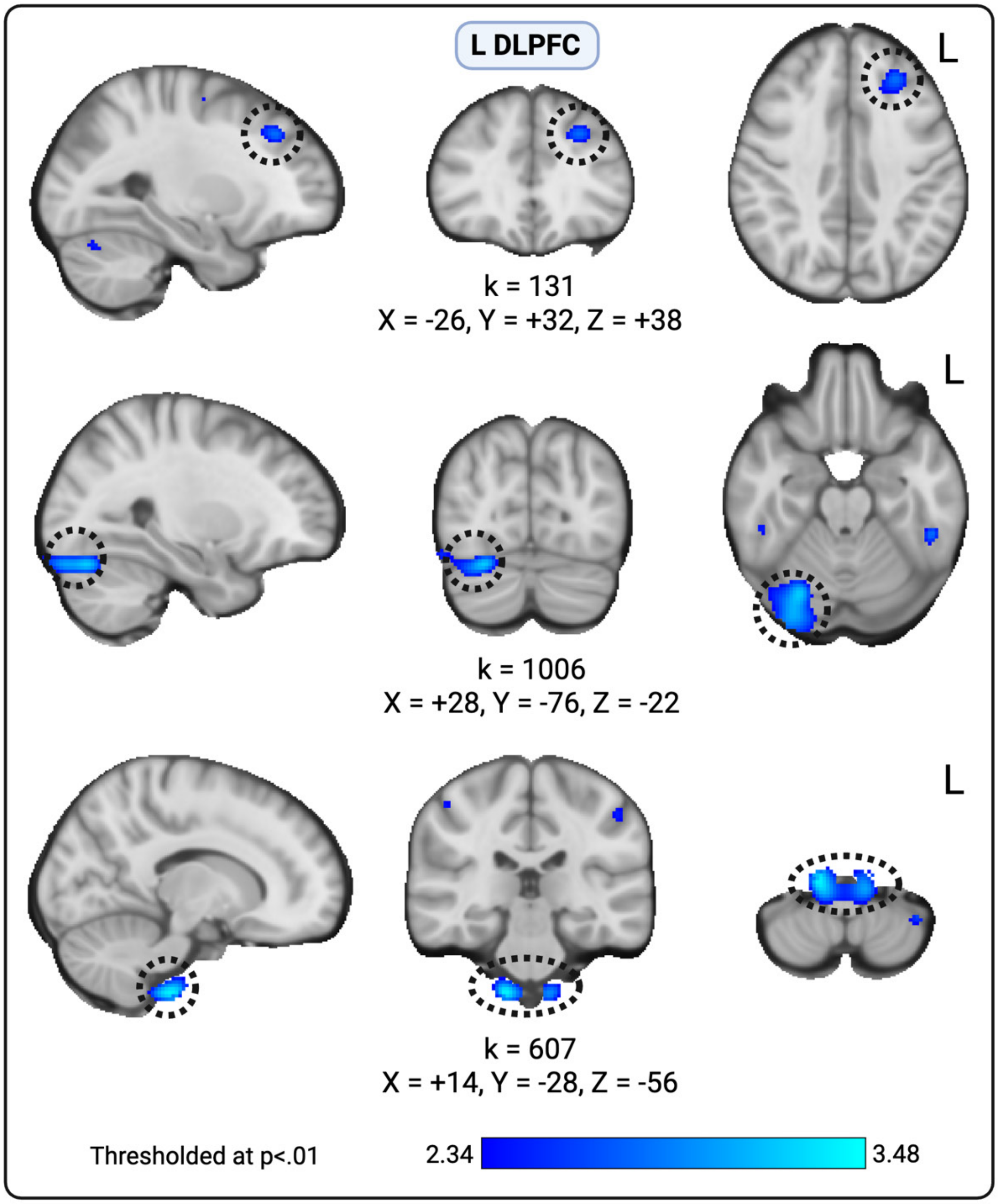

Supplemental Figure 3. In Unrestricted, Brain-wide Analysis, Cerebellar-DLPFC Connectivity is Associated with Negative Symptom Severity Across the Psychosis Spectrum. When we used the cerebellar region from Brady et al., 2019 as a seed and regressed brain-wide connectivity against negative symptom severity, controlling for age, sex, and scanner in our psychosis-spectrum sample (n=260), we observed a significant cluster k=131 centered at MNI X= -26, Y= +32, Z= +38 ($p<.01$). There were two other larger clusters identified in the right posterior cerebellum (k = 1006 at MNI X = +28, Y = -76, Z = -22) and brainstem (k=607 at MNI X = +14, Y = -28, Z = -56).

References

1. First MB, Frances A, Pincus HA. DSM-IV handbook of differential diagnosis. 1st ed. Washington, DC: American Psychiatric Press; 1995. xv, 247 p. p.
2. First M, Williams J, Karg R, Spitzer R. Structured Clinical Interview for DSM-5—Research Version (SCID-5 for DSM-5, Research Version; SCID-5-RV). Arlington, VA: American Psychiatric Association; 2015.
3. Kay SR, Fiszbein A, Opler LA. The positive and negative syndrome scale (PANSS) for schizophrenia. Schizophr Bull. 1987;13(2):261-76.
4. Marder SR, Davis JM, Chouinard G. The effects of risperidone on the five dimensions of schizophrenia derived by factor analysis: combined results of the North American trials. J Clin Psychiatry. 1997;58(12):538-46.
5. Jang SK, Choi HI, Park S, Jaekal E, Lee GY, Cho YI, et al. A Two-Factor Model Better Explains Heterogeneity in Negative Symptoms: Evidence from the Positive and Negative Syndrome Scale. Front Psychol. 2016;7:707.
6. Nadesalingam N, Kyrou A, Chapellier V, Maderthaner L, von Känel S, Wüthrich F, et al. Testing a Motor Score Based on PANSS Ratings: A Proxy for Comprehensive Motor Assessment. Schizophr Bull. 2024.
7. Montgomery SA, Asberg M. A new depression scale designed to be sensitive to change. Br J Psychiatry. 1979;134:382-9.
8. Brady RO, Jr., Gonsalvez I, Lee I, Ongur D, Seidman LJ, Schmahmann JD, et al. Cerebellar-Prefrontal Network Connectivity and Negative Symptoms in Schizophrenia. Am J Psychiatry. 2019:appiajp201818040429.
